# Delamination Mode I Analysis on Thin Stitch Fiberglass Composite

**DOI:** 10.3390/polym18050572

**Published:** 2026-02-27

**Authors:** Manuel Alejandro Lira-Martínez, Marianggy Gomez, Delfino Cornejo-Monroy, Jose Omar Davalos, Luis Asunción Pérez-Domínguez

**Affiliations:** Instituto de Ingeniería y Tecnología, Universidad Autónoma de Ciudad Juárez, Ciudad Juárez 32310, Mexico; marianggy.gomez@uacj.mx (M.G.); delfino.cornejo@uacj.mx (D.C.-M.); jose.davalos@uacj.mx (J.O.D.); luis.dominguez@uacj.mx (L.A.P.-D.)

**Keywords:** delamination, stitch materials, composite materials, composite laminates, interlaminar reinforcement, through-thickness stitching, Mode-I fracture, polyethylene thread, epoxy infusion, fiberglass

## Abstract

Delamination is a major failure Mode in laminated composites, typically triggered by premature interlaminar matrix cracking and leading to severe structural degradation. To address this, various through-thickness reinforcement strategies have been explored, including three-dimensional woven architecture. Although these designs significantly improve delamination resistance, their industrial adoption stays limited due to reproducibility challenges and the high cost and operational complexity of advanced manufacturing systems needed for controlled through-thickness reinforcement. This study investigates an alternative interlaminar reinforcement method, through-thickness stitching, aimed at enhancing Mode-I delamination resistance of a commercial fiberglass laminate without changing its native architecture. Composites were manufactured using a low-viscosity epoxy infusion system (MAX 1618 A/B) and a [0/90] biaxial fiberglass fabric. An eight-filament polyethylene thread (Ø = 0.12 mm) was introduced in predefined stitch architectures consisting of three longitudinal patterns having two, three, and five continuous stitch lines, referred to as AV, BV and CV samples, respectively. Results show that stitching highly increases Mode-I interlaminar fracture toughness G_IC_ by 0.3808, 0.4152 and 0.5192 kJ/m^2^ for AV, BV and CV respectively, compared to 0.0265 kJ/m^2^ for the unstitched composite O, highlighting the strong influence of stitch orientation and spacing on interlaminar performance. But scanning electron microscopy revealed added failure mechanisms in stitched specimens, including localized fiber misalignment of up to 33° and resin-rich regions approximately 0.6 mm in length, suggesting that while stitching enhances delamination resistance, it may also influence other mechanical properties.

## 1. Introduction

Composite materials are engineered multiphase systems produced by combining two or more distinct constituents to achieve properties that cannot be obtained from the individual components alone. In their typical architecture, the reinforcement phase bears and transfers mechanical loads, while the matrix phase surrounds, supports, and protects the reinforcement while contributing to the composite’s overall stiffness and dimensional stability [1,2]. A wide range of reinforcements—such as particles, continuous fibers, and discontinuous fibers—and matrix materials, including ceramics, metals, and polymers, can be employed; the specific choice and arrangement of these constituents ultimately determine the mechanical, thermal, and functional performance of the composite material [3,4].

Among the broad family of composite materials, polymer-matrix laminated composites reinforced with continuous fibers are one of the most widely used classes. These composites are typically manufactured by stacking two-dimensional textile plies, commonly composed of glass or carbon fibers arranged in axial or biaxial orientations, after impregnating them with a thermoset polymer matrix such as polyester or epoxy resin. The resulting laminate provides high in-plane stiffness and strength due to the continuous fiber architecture, while the matrix ensures load transfer, environmental protection, and structural integrity [5].

Composite materials play a critical role in the development of lightweight and high-performance structures across mechanical, aeronautical, and civil engineering applications. Their increasing use in place of metallic alloys is largely attributed to their superior specific strength and stiffness, along with enhanced corrosion resistance [6]. For example, a typical carbon fiber/epoxy laminate shows a density of approximately 1.8 g/cm^3^, which is substantially lower than that of common engineering metal alloys such as aluminum (2.7 g/cm^3^), steel (7.8 g/cm^3^), and titanium (4.5 g/cm^3^) [7]. This significant reduction in mass, combined with tailored mechanical performance, makes polymer-matrix composites highly attractive for weight-sensitive structural applications [8].

Despite their well-known advantages, laminated fiber-reinforced composites stay vulnerable to delamination, a critical failure Mode characterized by the separation of adjacent plies [9]. Delamination may be initiated under various conditions, including

- Direct or indirect loading applied to the interlaminar region, which is inherently weak due to the absence of reinforcing fibers [10].
- Environmental exposure, such as moisture ingress, extreme temperature fluctuations, or abrasive service conditions [11,12].
- Manufacturing-related imperfections, particularly matrix processing deficiencies that lead to entrapped air voids and, consequently, reduced mechanical performance [13].

Because delamination severely compromises the mechanical integrity of laminated composites, it can ultimately precipitate catastrophic structural failure [14]. As a result, the development of strategies to improve interlaminar performance continues to be a central focus of composite materials research. Current approaches include particle-reinforced matrices, interlaminar toughening plies, and three-dimensional reinforced composite architectures (MC3D) [15,16,17]. Among these, MC3D is the only configuration currently implemented in commercial applications—most notably by Safran—although its production remains costly due to the dependence on Jacquard weaving systems. Therefore, finding and evaluating alternative manufacturing routes for MC3D structures remains a critical objective.

Interlaminar fracture toughness, typically characterized by the Mode I strain energy release rate G_1C_ (kJ/m^2^), is a fundamental parameter for assessing a composite’s resistance to delamination [18]. Table 1 shows a comparison of G_1C_ values for conventional aerospace-grade laminated composites and several MC3D architectures, highlighting the markedly enhanced delamination resistance offered by three-dimensional reinforced systems.

Recent studies on MC3D composites have further underscored both their advantages and the challenges that remain. Hassan [32] reported that MC3D architectures manufactured using Jacquard looms exhibit residual stresses generated by yarn crimp and interlacing, which in turn reduce mechanical strength; however, partial stress mitigation was achieved through vacuum infusion processing. Gu [33] evaluated several Jacquard-woven configurations and observed friction-induced fiber damage that varied with fiber type and tow size while also noting that vacuum infusion facilitated the transfer of fiber-scale damage into the matrix. Pankow [34] identified premature matrix cracking associated with localized residual stress concentrations and excessive resin accumulation in void-like regions formed around inserted Z-reinforcements. Although these through-thickness reinforcements improved delamination resistance relative to conventional laminated composites, overly high Z-fiber content diminished overall mechanical performance. Monali [35] demonstrated that MC3D systems provide enhanced interlaminar shear resistance and enable fabrication in curved molds; however, Z-reinforcements may distort the in-plane fabric architecture during curing and can reduce delamination toughness by up to 20%. Hosseini et al. [5] further identified performance reductions resulting from irregular resin flow caused by the interconnection of the three orthogonal yarn systems. Callus et al. [36] reported a decreased elastic region during tensile loading, attributed to friction between the added reinforcement direction and the warp–weft system, as well as localized yarn entanglement during deformation.

The literature suggests several key guidelines for optimizing MC3D performance:

- Z reinforcements should be kept as thin as possible to minimize yarn crimp and the associated stress concentrations.
- Vacuum infusion has proven to be the most effective manufacturing route for reducing residual stresses in Z-reinforced architectures.
- Epoxy systems remain the preferred matrix due to their favorable mechanical compatibility with common reinforcement fibers.
- Among MC3D configurations, the orthogonal (ORT) architecture appears to be the most viable, as it exhibits minimal interlacing between warp and weft yarns, thereby reducing mechanically detrimental interactions.

Although recent studies have demonstrated the potential of MC3D architecture to significantly improve delamination resistance, several critical challenges must still be addressed to enable their widespread industrial adoption. As noted above, the reproducibility of these materials remains limited, principally due to the high cost and operational complexity of Jacquard weaving systems and other advanced manufacturing technologies required to produce controlled through thickness reinforcement.

Building on the challenges identified in previous studies and the need for scalable, cost-effective alternatives to MC3D architectures, the present research investigates the enhancement of interlaminar fracture toughness using a commercially available 2D woven fiberglass fabric reinforced through the thickness via stitching. As the stitching medium, a Superstrong PE Braided Fishing Line (4/12) was selected due to its small diameter and high tensile strength, making it suitable for through-thickness reinforcement without excessively disturbing the base fabric architecture. This strategy is motivated by the requirement to employ low-diameter orthogonal reinforcements that minimize distortion of the original 2D weave and preserve its in-plane mechanical properties. To assess the feasibility of this approach, three stitching patterns were designed and experimentally evaluated with the objective of increasing delamination resistance while limiting any detrimental effects on mechanical properties.

## 2. Materials and Methods

USA Fiberglass Warehouse Style 3733, a 3K biaxial [0/90] woven fabric, was selected as the primary reinforcement due to its commercial availability, adequate mechanical performance for structural applications, and a filament gauge that supports stable Z stitch insertion. Its plain weave architecture is particularly compatible with through-thickness reinforcement, allowing the stitching needle to penetrate the laminate with minimal disturbance to the in-plane fibers. The principal properties of this reinforcement are summarized in Table 2.

To ensure consistent impregnation of the stitched preforms, vacuum infusion was adopted as the manufacturing route. This process promotes uniform resin flow, reduces void formation, and has been shown to mitigate residual stresses associated with orthogonal Z stitching. The resin system selected for infusion was USA epoxy MAX 1618 A/B, chosen for its low mixed viscosity of approximately 377 cPs, ideal for infusion, along with its favorable mechanical characteristics (Table 3), compatibility with both reinforcement fabrics and stitch yarn, and ready commercial availability.

For the through-thickness reinforcement, a USA Superstrong PE Braided Fishing Line 4/12 was used. This four strand polyethylene braid offers a small diameter, high tensile strength, and good flexibility, characteristics that facilitate stitch insertion while minimizing local fiber waviness. Its low adhesion to epoxy also helps prevent undesirable resin perturbations, enabling the formation of a stable and mechanically efficient orthogonal stitching pattern. Available properties are shown in Table 4.

**Figure 1:**
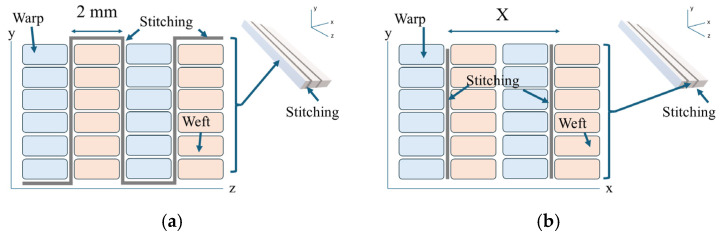
OW–TT stitch configuration: (**a**) transverse section; (**b**) longitudinal section (2 mm stitch pitch; transverse spacing = X).

An orthogonal weave with total-thickness reinforcement (OW–TT) configuration was selected based on prior analysis, which indicated that this arrangement minimizes mechanical degradation relative to other stitch orientations by reducing residual stresses arising from yarn–fiber friction and limiting the formation of resin-rich regions. The longitudinal stitch pitch was fixed at 2 mm, as shown in Figure 1a, in order to register with the underlying 3K warp–weft grid whose thread count is ~4 ends/cm (≈2.5 mm tow pitch) in the fiberglass fabric chosen, so a 2 mm stitch pitch places the needle path within or adjacent to a tow channel instead of repeatedly penetrating tow bundles, thereby reducing fiber waviness initiation sites. The transverse stitch spacing (denoted as X in Figure 1b) was systematically varied among samples to evaluate how stitch density influences both mechanical behavior and Mode I fracture response. The 2 mm longitudinal pitch was selected to register with the underlying 3K warp–weft grid whose thread count is ~4 ends/cm (≈2.5 mm tow pitch) in the fiberglass fabric chosen, so a 2 mm stitch pitch places the needle path within or adjacent to a tow channel instead of repeatedly penetrating tow bundles, thereby reducing fiber waviness initiation sites.

These samples are called AV, BV and CV, while a fourth sample O with no stitching will be elaborated for comparison purposes.

**Figure 2:**
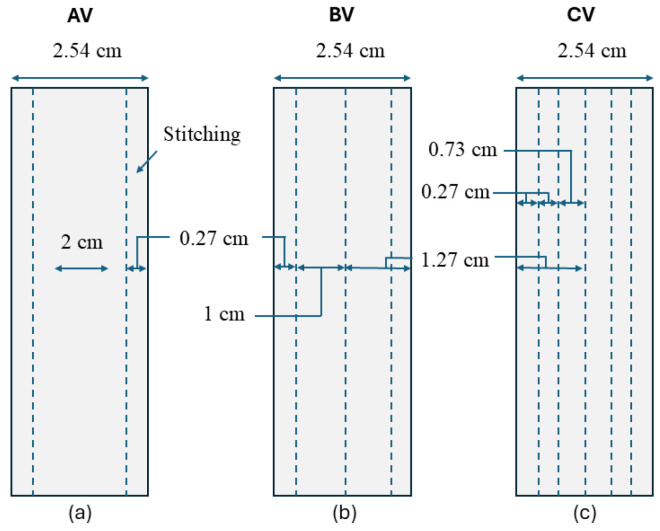
Longitudinal stitch patterns: (**a**) AV sample; (**b**) BV sample; (**c**) CV sample.

AV, BV, CV and O samples are shown in Figure 2 and were designed for specimens with a gauge length of 25.4 mm (1 inch), consistent with standard ASTM D5528 specimen tolerances [40]. Longitudinal orientation in the stitching across the specimen was selected because both tensile loading and Mode I delamination occur longitudinally. The characteristics of each stitching pattern are as follows:

- AV: stitches concentrated near the ends, where delamination typically initiates.
- BV: stitches concentrated at the center, addressing possible central energy localization.
- CV: stitches distributed across most of the gauge length.

Specimens were prepared following the dimensional and specific requirements of delamination Mode I standard ASTM D5528 [40], incorporating a 1-inch hinge length and an additional 50 mm allowance to introduce a pre-crack starter insert at mid thickness. Each sample was replicated in 5 specimens as shown in Table 5.

**Figure 3:**
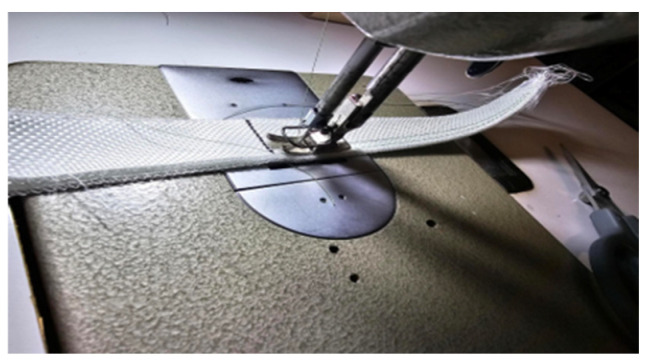
Stitching on Mercury MZ 20 43 sewing machine (DP × 5 needle, 2 mm).

Every laminate specimen was fabricated with six laminates of fiber glass, with a nominal thickness of ≈3 mm. Stitching was made with an industrial sewing machine, USA Mercury MZ 20 43, with a DP × 5 needle and 2 mm stitch length (as per the OW–TT pattern), as shown in Figure 3.

After stitching, edges were trimmed, dimensions were verified, and each specimen was weighed prior to infusion for fiber/matrix volume fraction calculations. After that, vacuum resin infusion was performed on a cleaned glass plate treated with mold release wax. The vacuum bag periphery was defined with double-sided infusion tape. Specimens were arranged, then covered with peel ply and flow mesh to facilitate uniform resin distribution. Inlet (distribution) and outlet (absorption) lines were installed at the top, followed by vacuum bag placement and sealing. A leak check (≥2 h hold) verified vacuum integrity. The epoxy (MAX 1618, 2:1 resin: hardener) was weighed, mixed for 5 min, degassed under vacuum, and infused at −27.5 inHg. Cure proceeded for 72 h; specimens were then demolded, polished, re-weighed, and prepared for testing.

Volume fraction was determined from specimen mass and geometric measurements, enabling estimation of the phase proportions in each laminate.

Mode I interlaminar fracture toughness was determined by the double cantilever beam (DCB) delamination test, according to ASTM D5528 [40], using 1-inch piano hinges to apply the load (F) through a CHN Jinan Heng Rui Jin WDW 100 universal testing machine (UTM). A vacuum-bagged laminate was used as a non-adhesive insert placed at the mid-plane to serve as the delamination initiator. The applied force and corresponding vertical displacement were recorded by the UTM, using a crosshead rate of 5 mm/min. All tests were video-recorded in 4K at 120 fps using a KOR Samsung Galaxy S24 Ultra smartphone with a green background. The footage was processed with Tracker version 6.3.3 software to determine the delamination length (a). Mode I interlaminar fracture toughness was calculated in Microsoft Excel using the Modified Beam Theory (Equation (1)). Results were plotted in OriginLab [40].

(1) $$G_{I}=\frac{3F\delta }{2ba}$$

where

F = Applied load, N;
δ = Load point deflection, mm;
b = Width of DCB specimen, mm;
a = Delamination length, mm.

Fracture mechanisms of failed delamination specimens were examined using a JAP Hitachi SU5000 SEM. The analysis included: mechanism classification; dimple (tow gap) diameters; resin-rich regions; stitch knot deformation; fiber/matrix fractures; fiber breaks near stitches; local matrix cracking near stitches; deformation of broken yarn tips; fiber displacement due to stitching; evidence of plastic deformation in resin, fibers, and stitch yarn; and internal fiber pull-outs/debonded white zones.

## 3. Results

### 3.1. Fiber/Matrix Volume Fraction

Table 6 summarizes the samples’ average fiber volume fraction (FVF), matrix volume fraction (MVF), and stitch volume fraction (SVF), by pattern. Target FVFs of 0.50–0.60 were achieved for all stitch specimens but were lower than the N sample, indicating a non-ideal constituent ratio that is expected to influence mechanical properties according to the rule of mixtures.

### 3.2. Delamination Test

**Figure 4:**
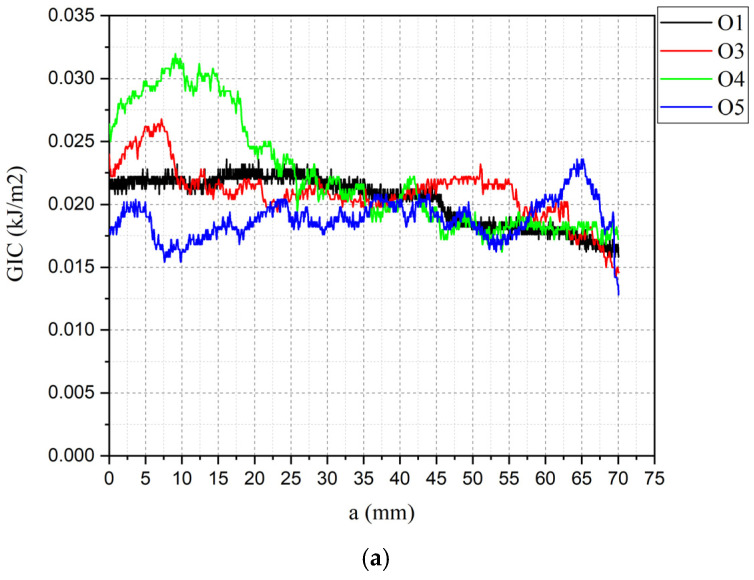

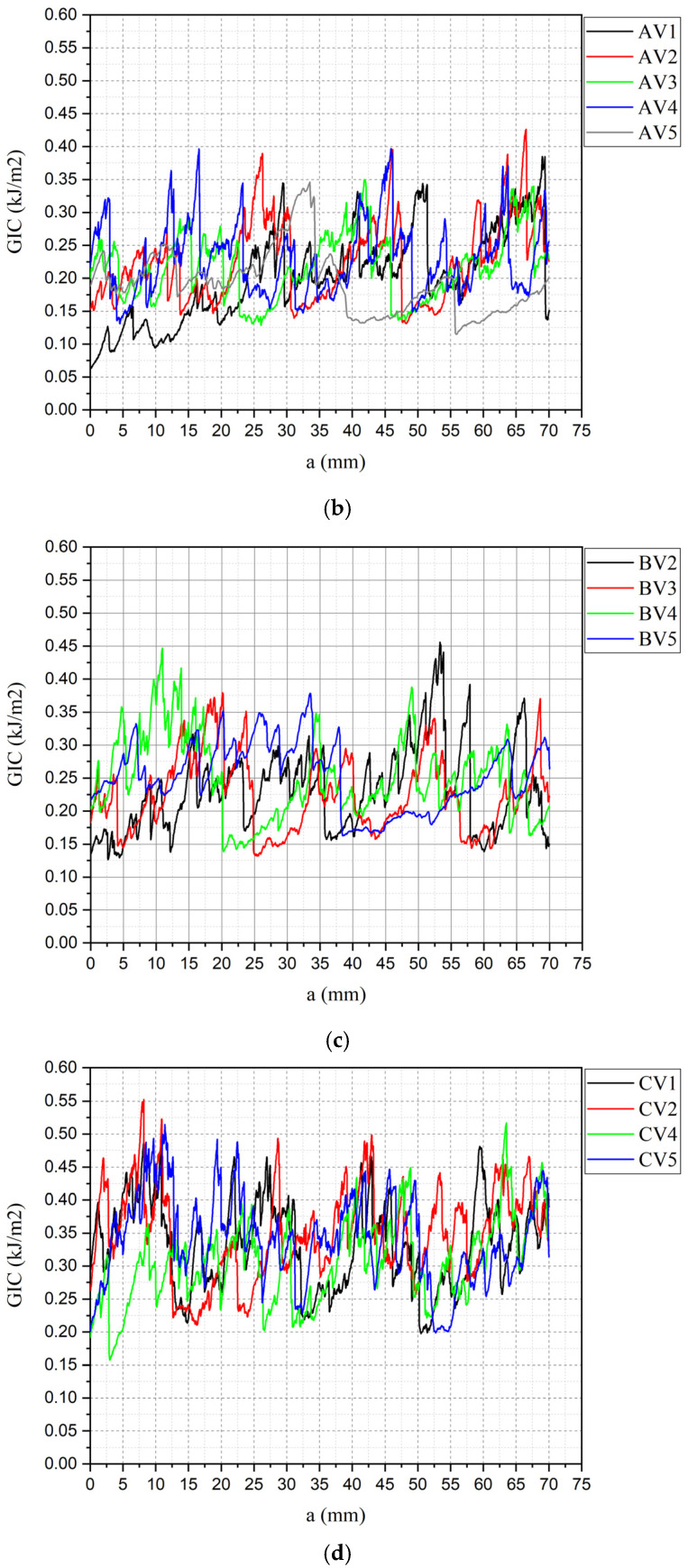
Mode I G_IC_–a curves: (**a**) O; (**b**) AV; (**c**) BV; (**d**) CV.

Delamination curves for each valid specimen group are presented in Figure 4, and Table 7 shows the maximum, mean and minimum specimen’s GI values. To demonstrate that differences between AV, BV and CV are statistically significant despite the small sample size, a pairwise Welch’s t test was done using Table 7 data. The results show that CV is significantly higher than both AV and BV, even after Holm correction for multiple testing: AV vs. CV t (6.98) = −7.12, pHolm = 0.00058; BV vs. CV t (4.85) = −4.26, pHolm = 0.0171. The contrast between AV and BV was not significant: t (5.72) = −1.33, pHolm = 0.235. Thus, these results confirm statistically significant differences among the samples.

Specimens result of O2, BV1 and CV3 were discarded because of premature failure in hinges. The unstitched control specimens (O) showed exceptionally low G_IC_, with a value of 0.0265 kJ/m^2^, consistent with typical Mode I fracture toughness values reported for laminated composites without interlaminar toughening. In contrast, the stitched groups displayed highly fluctuating and spatially dispersed profiles, characteristic of repeated cycles of load accumulation and release as individual stitches fractured during crack propagation. Stitched samples had a considerable increase in G_IC_, following the trend AV < BV < CV, showing that increasing stitch density generally enhances interlaminar toughness as the values were 0.3808, 0.4125 and 0.5192 kJ/m^2^, respectively. In the AV pattern, stitches were concentrated near the specimen ends, offering localized crack bridging only at early crack growth stages. As a result, once the first reinforcement points were fractured, the delamination front progressed with limited added resistance. The BV pattern, which concentrates stitches near the mid length, provided a more sustained toughening effect due to its positioning closer to regions where energy release rates tend to intensify during propagation. The CV configuration, incorporating stitches distributed along most of the gauge length, produced the highest Mode I resistance, as the delamination front met a larger number of through-thickness constraints throughout the crack path. This broader spatial distribution of stitch bridging sites increased both crack deflection opportunities and the number of load transfer events before individual stitch failure, thereby elevating overall interlaminar toughness. Collectively, these results confirm that greater stitch density and spatial coverage enhance crack bridging efficiency, providing a more robust barrier against delamination propagation.

**Figure 5:**
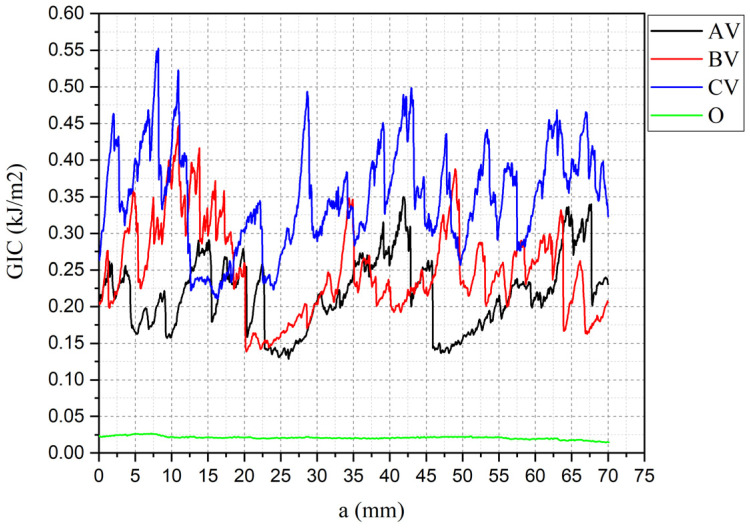
Representative delamination curve overlay of all average samples’ curves.

**Figure 6:**
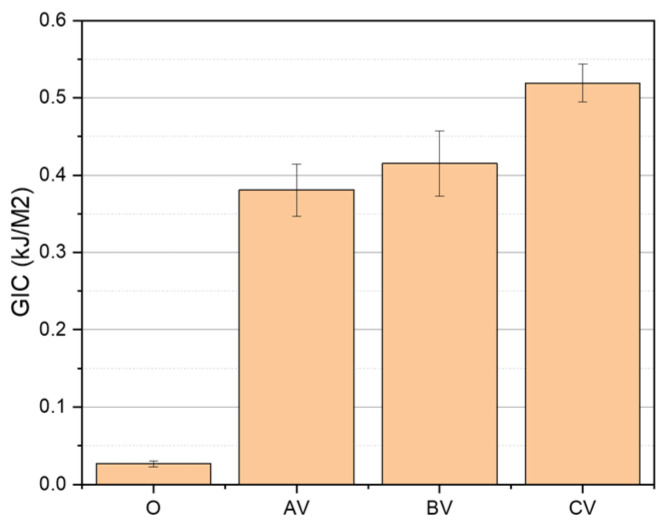
Bar plot of average G_IC_ for AV, BV, CV vs. O.

A representative delamination curve overlay shown in Figure 5 further highlights the substantial gains from stitching compared to the unstitched sample (O). The average G_IC_ increased by +1437.0% (AV), +1566.6% (BV), and +1959.2% (CV) compared with O (see Table 8 and Figure 6). Moreover, even the minimum G_I_ values for all stitched samples exceeded the G_IC_ of O sample, corroborating the robustness of the toughening effect across the crack growth range.

**Figure 7:**
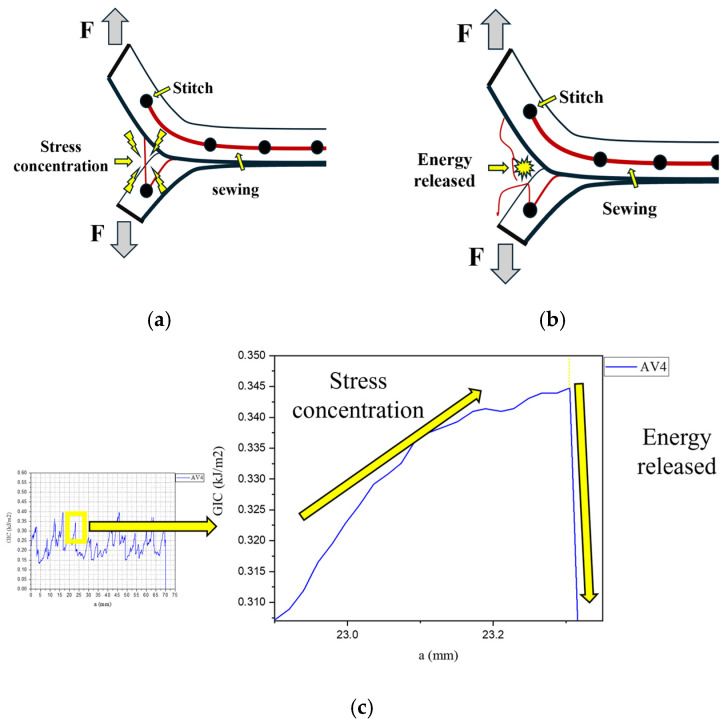
Fracture mechanism of stitching in stitched specimens: (**a**) Stress concentration due to stitch toughness, (**b**) stitch fracture and energy release, (**c**) mechanisms that generate peaks in delamination curve.

The mechanics underlying the fluctuations within the delamination curves of stitched samples are illustrated in Figure 7. These oscillations arise primarily from the high energy required to sequentially fracture the warp stitches along the specimen (Figure 7a). Following each stitch failure, a drop in energy is observed due to stress release from the fractured stitch (Figure 7b) until the crack front reaches the next stitch, thereby repeating the cycle and producing the oscillatory pattern shown in Figure 7c [41,42,43].

**Figure 8:**
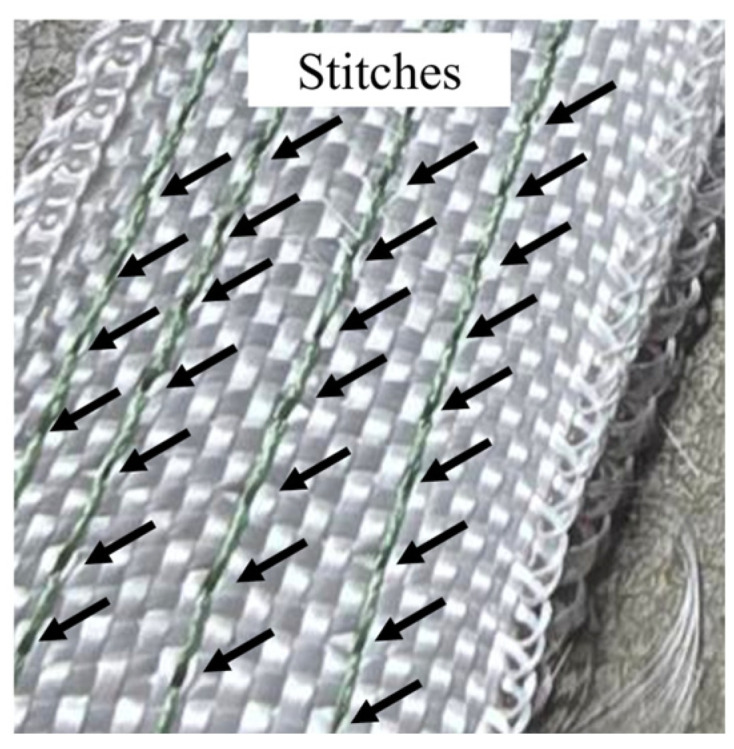
Asymmetric stitches in BV specimen.

It is worth noting that the continuous warp stitch lines along the specimen are not perfectly symmetric across adjacent seams. For example, in the BV specimens, comprising four continuous seams, the stitch locations on one side do not align exactly with those on the opposite side (Figure 8). Consequently, the stress distribution is also asymmetric, which can influence local crack-bridging events and the sequence of stitch engagement during delamination growth.

**Figure 9:**
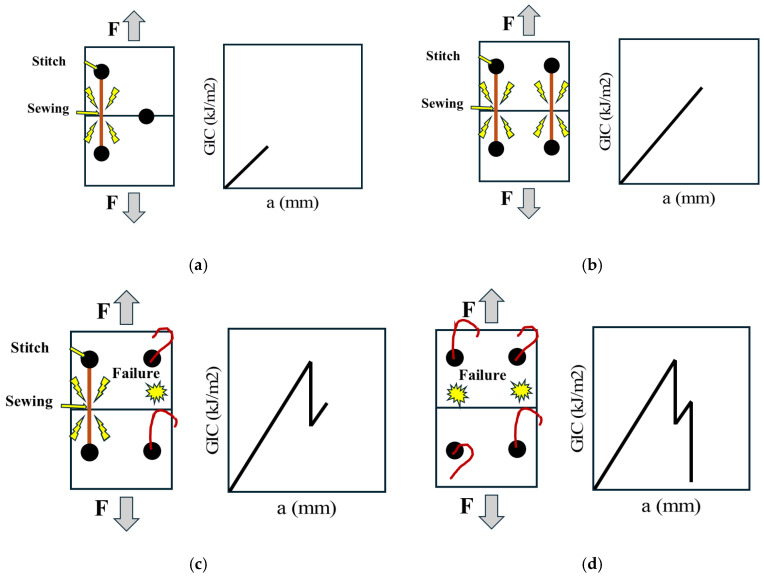
Mechanism for generating peaks of different magnitudes through the combined effect of two stitches: (**a**) Energy generated due to a single stitch, (**b**) increase in applied energy when loading engages two stitches, (**c**) energy released and partial recovery caused by the failure of one stitch, (**d**) energy released associated with the failure of both stitches.

This behavior yields the mechanism illustrated in Figure 9. Along with the specimen length, the stress field generated by one stitch may overlap with that of one or more adjacent stitches (Figure 9a), producing energy concentrations that manifest as multiple peaks of varying magnitudes; these can superimpose, creating peaks of higher amplitude than their individual counterparts (Figure 9b). The elevated stress is then released as the stitches fracture; however, this release can be progressive rather than instantaneous. For example, a stitch may fail in the vicinity of another stitch that remains engaged (Figure 9c), creating a valley in the response while maintaining a localized stress concentration around the nearest intact stitch until it, too, fails and the corresponding energy is released (Figure 9d). Consequently, greater stitch adjacency (higher local stitch density) produces peaks of higher magnitude and frequency, explaining the proportionality between increased stitch volume and higher interlaminar toughness, and the observed ranking wherein AV < BV < CV for G_IC_ [44,45,46].

**Figure 10:**
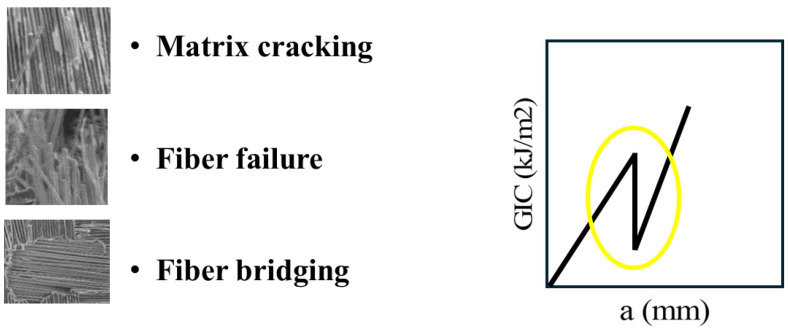
Other mechanisms that modify the energy kinetics in the delamination curve.

Other damage mechanisms that contribute to the appearance of load drops and to the modification of the energy release kinetics—producing a “splinter-type” response or localized energy discontinuities in the delamination diagram—include matrix cracking, fiber–matrix debonding, fiber bridging and fiber failure. These mechanisms arise from stresses that are not fully absorbed by the stitches and manifest in the delamination curves as small peaks and saw patterns, as illustrated in Figure 10. Although these events generate lower energy values compared to those associated with stitch rupture, they nevertheless contribute to the overall energy concentration and to the governing failure mechanics of the composite [47,48].

**Figure 11:**
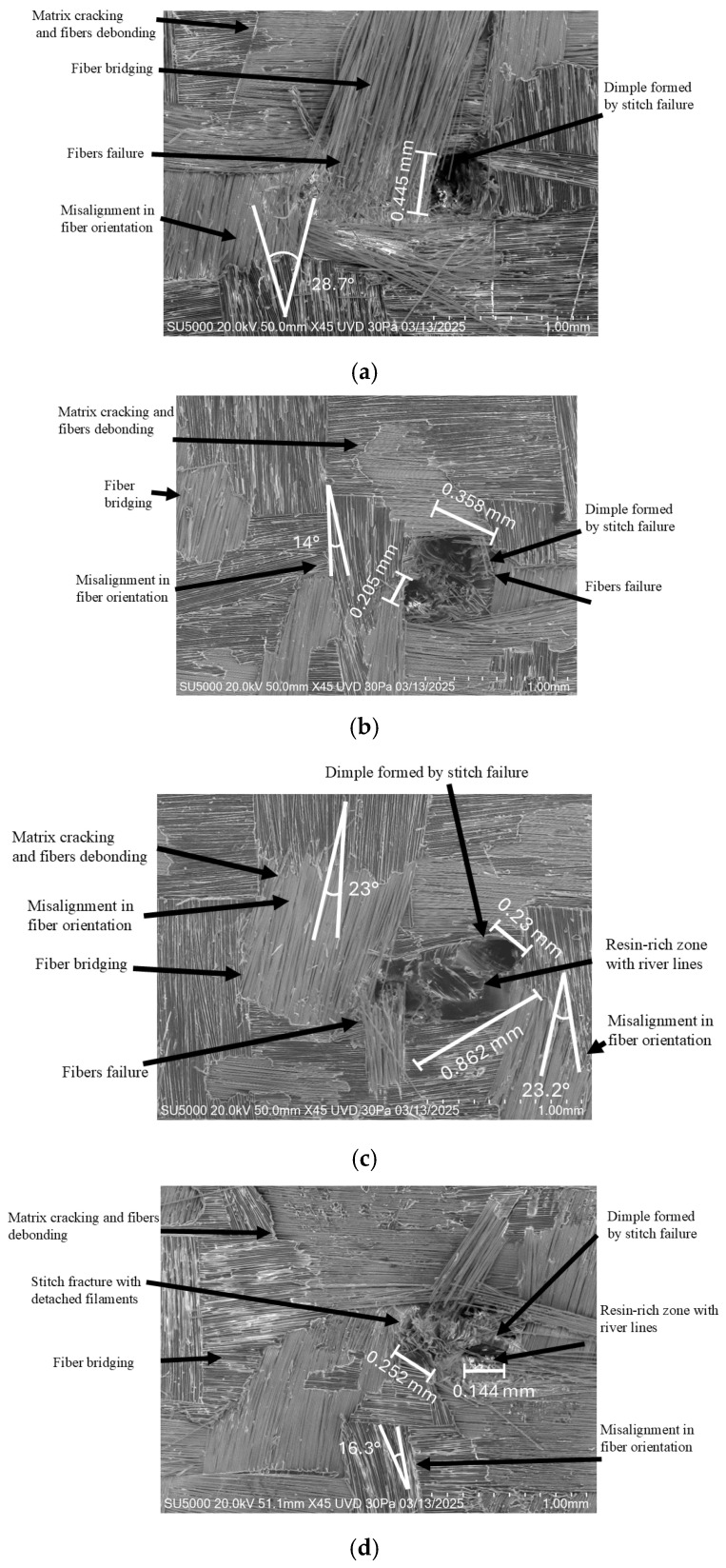
Microscopy of fractured CV specimen: (**a**) central section, (**b**) central section, (**c**) edge section, (**d**) edge section.

Microscopy and post-failure analysis of the stitched specimens fractured under delamination revealed several fracture mechanisms, which are illustrated in Figure 11:

- Matrix cracking: Brittle fracture of the polymer matrix caused by the redistribution of energy toward and away from the fibers and the stitching, leading to local detachment phenomena [47,49,50].
- Fiber bridging: Groups of fabric fibers that remained partially attached and were lifted out of the fracture plane due to resin cracking [9,51].
- Fiber failure: Broken fibers located around the stitch threads or stitch-induced dimples, produced by transverse stress concentrations generated during the test as the stitches redistributed interlaminar loads [52,53].
- Stitch-induced dimples: Local cavities associated with the extraction of fractured stitches pulled out by the parallel stitch counterpart, with different ranges from 0.2 to 0.445 mm (Figure 11a) in diameter [49,51].
- Partially fractured stitches with detached filaments: Stitch threads exhibiting incomplete rupture with several loose or separated yarn filaments.
- Fiber misalignment: Disturbance of the original biaxial fiber orientation, reaching deviations of up to 28.7° (Figure 11a), resulting from interlaminar stresses in the vicinity of the stitches, in some cases producing localized waviness [33,52,53,54,55].
- Resin-rich zones with river lines and cleavage steps near the stitches, extending longitudinally up to 0.862 mm. (Figure 11c): Resin-rich regions surrounding the stitch perimeter exhibiting brittle fracture patterns, including cleavage steps and river line features, resulting from the absorption and subsequent redistribution of interlaminar energy toward the stitch threads [56,57].

These mechanisms were observed abundantly in all stitched specimens, in agreement with the fluctuations recorded in the delamination diagrams. A higher concentration of matrix-related fracture regions was identified as the stitch density increased. It was also noted that, as the specimens progressed from the pre-failure stage toward complete fracture, matrix cracking and fiber-related damage intensified, whereas stitch-induced dimples became less prevalent.

Additional mechanisms—such as resin-rich zones surrounding the stitch dimples and distortions in fiber orientation—were also detected. Although these features did not significantly influence the overall energy release kinetics, given the absence of fiber breakage within the waviness zones and the relatively small cleavage steps and river line markings, their presence is noteworthy. These microstructural irregularities act as stress concentrators that may weaken the material and potentially compromise other mechanical properties, including compressive strength, flexural performance, fatigue resistance, and susceptibility to different delamination Modes, by altering load transfer conditions between the reinforcement and matrix [9,33,50,51,52,53].

**Figure 12:**
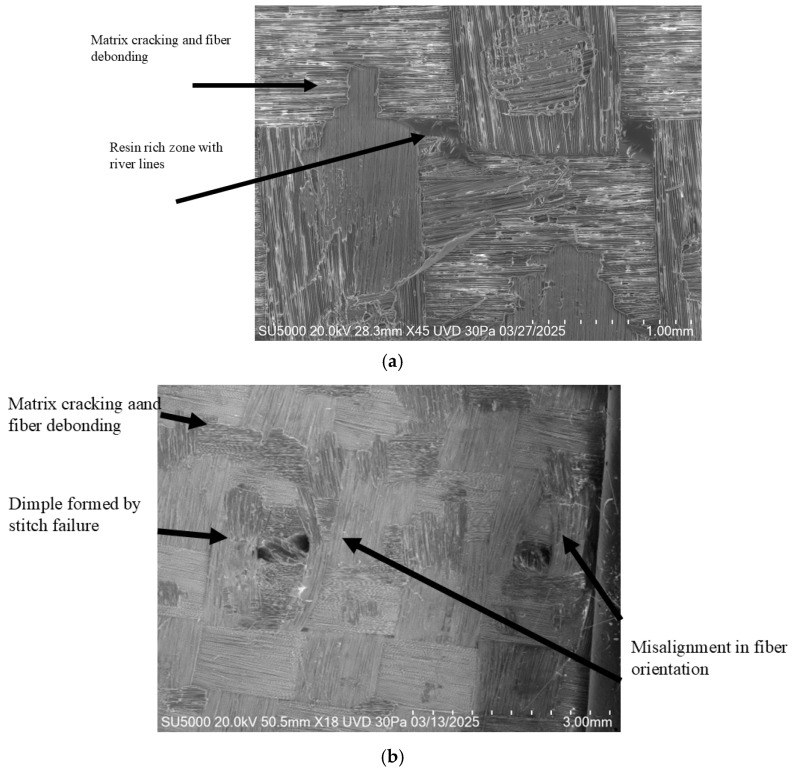
Comparison of specimen without stitching vs. specimen with stitching: (**a**) Matrix fracture and resin-rich regions with river lines, (**b**) matrix cracking and fiber debonding, dimples, and misalignment in fiber orientation.

Figure 12a shows images of O specimens, which exhibit only minimal and simple fracture mechanisms such as matrix cracking, resin-rich areas, and limited fiber debonding. In contrast, stitched specimens present more complex and pronounced damage features—stitch-induced dimples, fiber orientation disturbances, extensive matrix fractures, resin-rich regions, and more evident fiber pull-out—as illustrated in Figure 12b.

## 4. Discussion

- When comparing the Mode I delamination resistance of the stitched specimens with values reported for MC3D composites in Table 1, the incorporation of polyethylene stitching into commercially available fiberglass/epoxy laminates demonstrated a simple yet effective strategy for improving interlaminar performance. All three longitudinal stitching patterns (AV, BV, and CV) have a significant increase in G_IC_, reaching 0.3808, 0.4152, and 0.5192 kJ/m^2^, respectively. These values fall within the broad range documented (0.101 kJ/m^2^ to 11.6 kJ/m^2^), with quasi-isotropic 3D carbon fiber/epoxy-reinforced with short Kevlar fibers [24] being the closest comparable G_IC_ with 0.421 kJ/m^2^.
- The present results align with multiple recent investigations showing that three-dimensional reinforcement significantly enhances delamination resistance and that higher z-reinforcement volume fractions yield greater interlaminar fracture toughness [33,35,43,44,46,51,52,56]. This trend was also confirmed experimentally: the CV pattern, with the highest reinforcement fraction, exhibited the largest G_IC_ value, followed by BV and AV. The literature further indicates that delamination behavior is highly dependent on the reinforcement architecture, stitch density, material system, and matrix–fiber interaction. The highest improvements in G_IC_ are typically achieved when a dense transverse reinforcement is introduced through the laminate thickness, effectively suppressing the influence of the in-plane fiber orientation.
- Despite the notable gains in interlaminar toughness, the addition of stitching induced the disruption of the in-plane fiber continuity by misalignment up to 33°, combined with the formation of resin-rich regions of approximately 0.6 mm in length and local fiber waviness, both widely recognized mechanisms that compromise load transfer efficiency, and thus mechanical properties might be reduced. For this reason, future studies of characterizing different mechanical properties of these samples must be done.
- The literature reports considerable variability regarding the influence of stitching on mechanical properties other than delamination [33,56,58]. Depending on stitch orientation, density, and fabrication technique, tensile strength may increase, remain unchanged, or decrease. Favorable outcomes have been reported for layer-to-layer (LL) and angle-interlock (AI) architectures, where the stitch alignment supports the direction of applied load [12,44,59]. In contrast, 2.5D stitching, due to interlacing across layers, may provide further improvements in tensile strength [12,26]. When minimizing tensile degradation is prioritized, straight, minimally intrusive stitches—such as OW and TT patterns—are recommended, together with vacuum infusion, to reduce residual stresses [27]. Nevertheless, even these methods can still induce fiber waviness and resin-rich zones, ultimately compromising mechanical properties such as compression strength, fatigue life, and flexural stiffness [60,61].
- Overall, the findings highlight the inherent balance required when integrating through-thickness reinforcement in laminated composites. While stitching provides a clear and substantial improvement in interlaminar fracture resistance, it inevitably modifies in-plane properties to some extent. Therefore, careful optimization of stitch density, orientation, diameter, and processing conditions is essential to achieving a desirable compromise between damage tolerance and structural integrity.

## Figures and Tables

**Table 1:** Comparison of Mode I interlaminar fracture toughness values (G_IC_, kJ/m^2^) for conventional laminated fiber-reinforced composites and various three-dimensional (MC3D) architectures reported in the literature.

Conventional Fiber-Reinforced Composites		
Reinforcement/Matrix System	G_1C_ (kJ/m^2^)	Reference
Biaxial fiberglass [0/90]/epoxy	0.01–0.03	[19]
Biaxial carbon fiber [0/90]/epoxy	0.02–0.04	[20]
Biaxial aramid fiber [0/90]/epoxy	0.03–0.05	[21]
Biaxial fiberglass [0/90]/polyester	0.005–0.01	[22]
Biaxial carbon fiber [0/90]/polyester	0.001–0.02	[23]
Biaxial aramid fiber [0/90]/polyester	0.015–0.025	[24]
Biaxial AS4 carbon fiber [0/90]/3502 resin	0.117	[25]
Biaxial AS4 carbon fiber [0/90]/1808 resin	0.231	[1]
MC3D Fiber-Reinforced Composites		
Reinforcement/Matrix System	G_1C_ (kJ/m^2^)	Reference
Quasi-isotropic 3D carbon fiber/epoxy with short Kevlar fibers	0.421	[26]
3D satin-woven carbon fiber/boron nitride	0.175	[27]
3D LTL carbon fiber fabric/epoxy reinforced with carbon nanoparticles	11.6	[28]
3D fiberglass fabric/epoxy reinforced with ethylene-vinyl acetate	0.101	[29]
3D ORT carbon fiber fabric/epoxy	4.688	[30]
3D fiberglass fabric/polyester with glass-particle reinforcement	1.359	[17]
1K carbon fiber 3D fabric/epoxy	0.823	[18]
AS4 carbon fiber/1808 resin	0.231	[31]

**Table 2:** Key properties of selected fiber reinforcement [37].

Property	Fiberglass Warehouse Style 3733
UTS (MPa)	3400
Modulus, E (GPa)	72–74
Density (g/cm^3^)	1.94
Strain at break (%)	2.2–2.8
Filament diameter (µm)	~9 µm
Gauge	3K

**Table 3:** Principal properties of the MAX 1618 epoxy system [38].

Property	MAX 1618 Epoxy Resin
Peel strength (kg/cm)	1.01
Shear strength (MPa)	13.1
Flexural strength (MPa)	89.63
Hardness (Shore D)	80 ± 5
Elongation (%)	3
Density (g/cm^3^)	1.09 ± 0.03
Viscosity (cPs)	377
Working time (min)	30–45
Cure time (h @ 25 °C)	≥36
Service temperature (°C)	95

**Table 4:** Stitch yarn properties [39].

Property	Superstrong PE Braided Fishing Line 4/12
UTS (MPa)	6.79
Diameter (mm)	0.12
Strands	4

**Table 5:** Specimen matrix for delamination tests.

Sample	O	AV	BV	CV
Specimen denomination	O1–O5	AV1–AV5	BV1–BV5	CV1–CV5

**Table 6:** Average volume fractions of samples.

Sample	FVF	SVF	MVF	SD
O	0.579	NA	0.42	0.025
AV	0.56	0.009	0.43	0.076
BV	0.548	0.015	0.438	0.035
CV	0.533	0.023	0.442	0.113

**Table 7:** Maximum, mean and minimum G_I_ specimen values from delamination test.

Sample	Specimen	G_IC_ (kJ/m^2^)	G_I_ Mean (kJ/m^2^)	G_I_ Min (kJ/m^2^)
O	O1	0.0236	0.0203	0.0158
	O3	0.0268	0.021	0.0142
	O4	0.032	0.0224	0.0162
	O5	0.0236	0.0189	0.0128
	Mean ± SD	0.0265 ± 0.0040	0.0206 ± 0.0014	0.0148 ± 0.0016
AV	AV1	0.3849	0.2024	0.0629
	AV2	0.4262	0.2251	0.1308
	AV3	0.3498	0.218	0.1283
	AV4	0.3971	0.2292	0.1308
	AV5	0.346	0.1938	0.1148
	Mean ± SD	0.3808 ± 0.0336	0.2137 ± 0.0151	0.1135 ± 0.0291
BV	BV2	0.456	0.2319	0.1265
	BV3	0.3792	0.2251	0.1319
	BV4	0.4469	0.2498	0.1387
	BV5	0.3785	0.2528	0.1623
	Mean ± SD	0.4152 ± 0.0421	0.2399 ± 0.0135	0.1398 ± 0.0158
CV	CV1	0.4929	0.3328	0.1972
	CV2	0.5525	0.349	0.2105
	CV4	0.5168	0.3066	0.1573
	CV5	0.5145	0.3408	0.1987
	Mean ± SD	0.5192 ± 0.0247	0.3323 ± 0.0184	0.1910 ± 0.0232

**Table 8:** Summary statistics and percentage gains vs. O for stitched samples.

Metric	O	AV	Δ% vs. O	BV	Δ% vs. O	CV	Δ% vs. O
G_IC_ (kJ/m^2^)	0.0265	0.3808	1437.02	0.4152	1566.6	0.5192	1959.15
G_I_ Mean (kJ/m^2^)	0.0206	0.2137	1036.11	0.2399	1163.2	0.3323	1611.12
G_I_ Min (kJ/m^2^)	0.0148	0.1135	769.61	0.1398	948.06	0.191	1294.57

## Data Availability

The original contributions presented in this study are included in the article. Further inquiries can be directed to the corresponding author.

## Abbreviations

The following abbreviations are used in this manuscript:

MC3D	Three-dimensional reinforced composite architectures
G_IC_	Critical energy release rate for Mode I delamination
G_I_	Energy release rate for Mode I delamination
SD	Standard deviation
t	Test statistic t
pHolm	Holm-adjusted p-value
UTS	Ultimate tensile strength
E	Modulus of elasticity
OW	Orthogonal weave
FVF	Fiber volume fraction
SVF	Stitch volume fraction
MVF	Matrix volume fraction
LL	Layer-to-layer
AI	Angle-interlock
F	Applied load
δ	Load point deflection
b	Width of DCB specimen
a	Delamination length
